# Cardiac tamponade due to rupture of a giant coronary artery aneurysm with a coronary arteriovenous fistula: a case report

**DOI:** 10.1186/s40792-019-0597-6

**Published:** 2019-03-12

**Authors:** Yu Shomura, Toru Mizumoto, Kazuya Fujinaga, Yasuhiro Sawada, Hisato Ito, Satoshi Teranishi

**Affiliations:** 1 Department of Thoracic and Cardiovascular Surgery, Anjo Kosei Hospital, 28 Higashihirokute, Anjocho, Anjo, Aichi 446-8602 Japan; 2 Department of Emergency Medicine, Anjo Kosei Hospital, 28 Higashihirokute, Anjocho, Anjo, Aichi 446-8602 Japan

**Keywords:** Cardiac tamponade, Giant coronary artery aneurysm, Coronary arteriovenous fistula

## Abstract

**Background:**

Coronary artery aneurysm (CAA) is defined as dilatation exceeding 1.5 times the width of the normal adjacent coronary artery segments. CAA usually causes few symptoms, and rupture is rare, but can be lethal due to cardiac tamponade when it does occur.

**Case presentation:**

A 79-year-old woman presented with presyncope and back pain. Emergency surgery was performed based on a diagnosis of cardiac tamponade due to either rupture of coronary arteriovenous fistula or CAA. At surgery, a rupture site was located on the wall of the giant CAA, with a diameter of 55 mm, originating from the ostium of the right coronary artery. Suture closure of the inflow and outflow of the aneurysm was performed, and the aneurysmal cavity was obliterated by multiple sutures. The patient made an uneventful recovery and was discharged from hospital on postoperative day 13.

**Conclusion:**

On the basis of this case, we propose considering rupture of a CAA as one of the causes of cardiac tamponade.

## Background

Coronary artery aneurysm (CAA) is defined as dilatation exceeding 1.5 times the width of the normal adjacent coronary artery segments. Those with a diameter > 20 mm are referred to as giant CAA [1]. CAA usually causes few symptoms, and rupture is rare, but can be lethal due to cardiac tamponade when it does occur.

We describe a patient with acute cardiac tamponade caused by spontaneous rupture of giant CAA with a coronary arteriovenous fistula (CAVF), who underwent successful emergency surgery.

## Case presentation

**Fig. 1:**
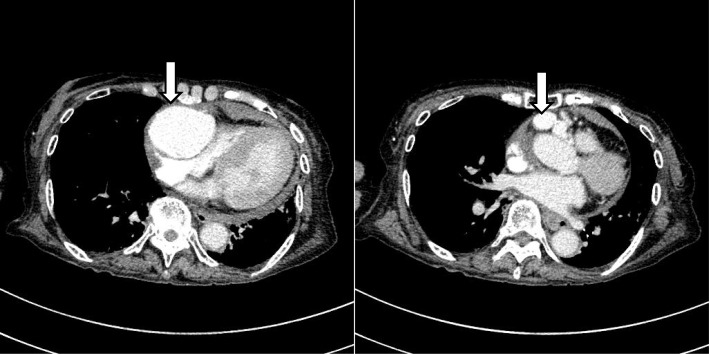
Initial computed tomography of the chest. Computed tomography of the chest shows multiple aneurysmal masses (arrows) in front of the heart communicating proximally with the right coronary artery

**Fig. 2:**
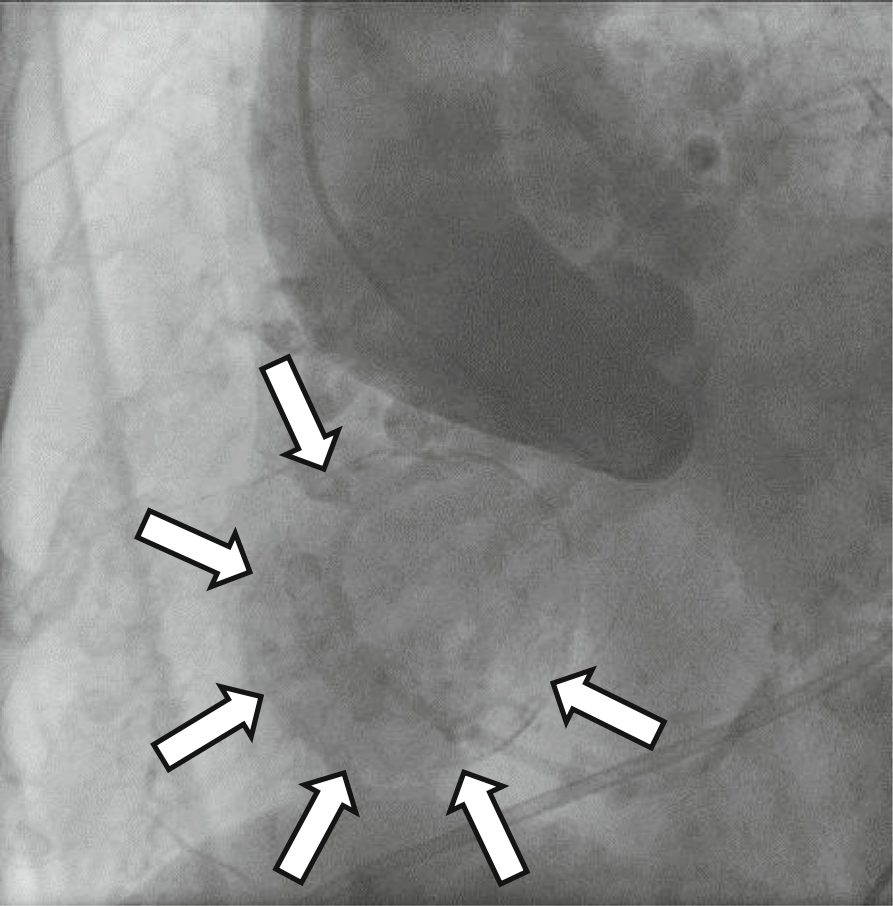
Coronary angiography. Coronary angiography reveals three coronary artery aneurysms with coronary arteriovenous fistulae. One of the aneurysms from the right coronary artery was a giant coronary artery aneurysm (arrow) with a diameter of 55 mm

A 79-year-old woman was transferred to a nearby hospital because of presyncope and back pain. She had no history of Kawasaki disease or prior thoracic trauma history. On arrival in the nearby hospital, her heart rate was 90 beats/min, and systolic blood pressure was 60 mmHg. Laboratory analyses showed elevated liver enzymes (glutamic oxaloacetic transaminase, 815 IU/L; glutamic pyruvic transaminase, 240 IU/L; lactate dehydrogenase, 924 IU/L), likely induced in response to shock. Transthoracic echocardiography revealed a 37 × 54-mm spherical mass adjacent to the right lateral atrioventricular groove and decompression of the right ventricle resulting from pericardial effusion. Systolic blood pressure improved to 100 mmHg after pericardial drainage (100 mL of blood) and infusion of a vasopressor. Computed tomography (CT) showed multiple aneurysmal masses in front of the heart communicating proximally with the right coronary artery (RCA) (Fig. 1). Coronary angiography was therefore performed, revealing three CAA with CAVF. Two of the aneurysms drained into the pulmonary artery and originated from a branch of the RCA, and another arose from the origin of the left anterior descending artery (LAD) and drained into the pulmonary artery. One of the aneurysms from the RCA was a giant CAA, with a diameter of 55 mm (Fig. 2).

**Fig. 3:**
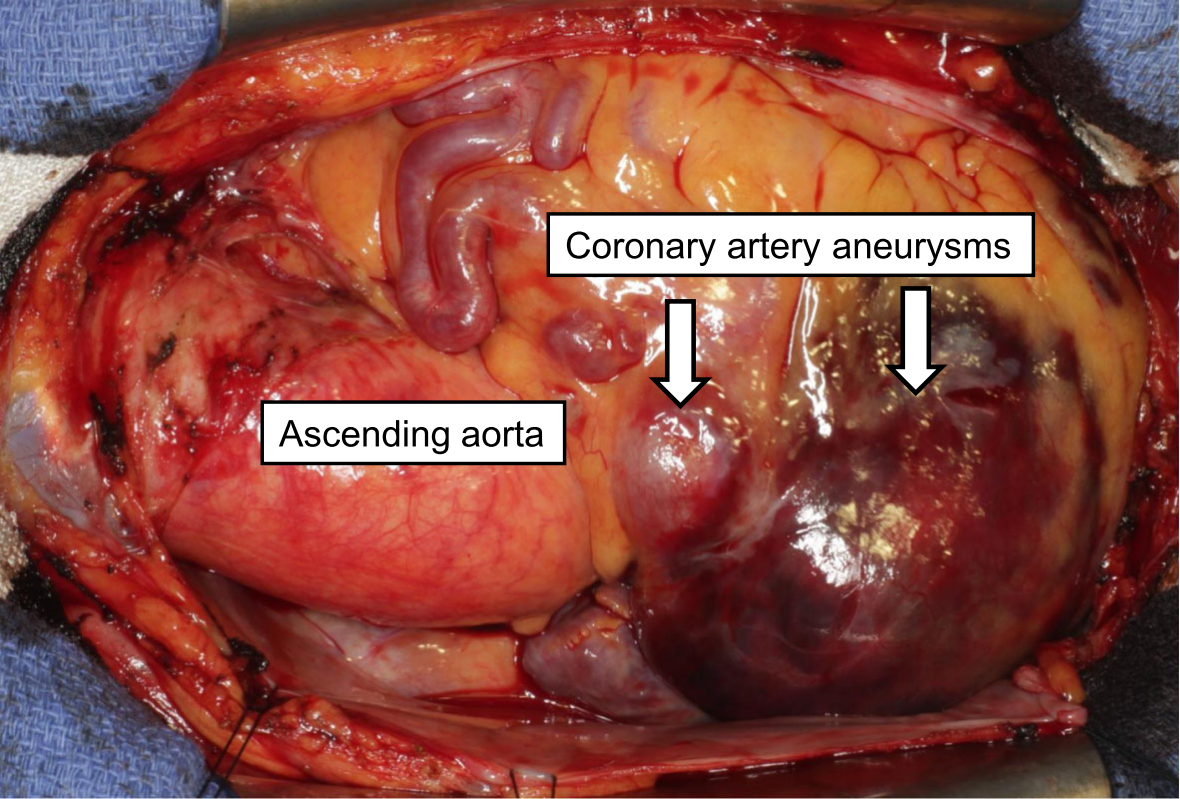
Intraoperative photograph. Aneurysms (arrows) originating from the ostium of the right coronary artery. Blood oozing is evident and a rupture site is located on the wall of the giant coronary artery aneurysm

**Fig. 4:**
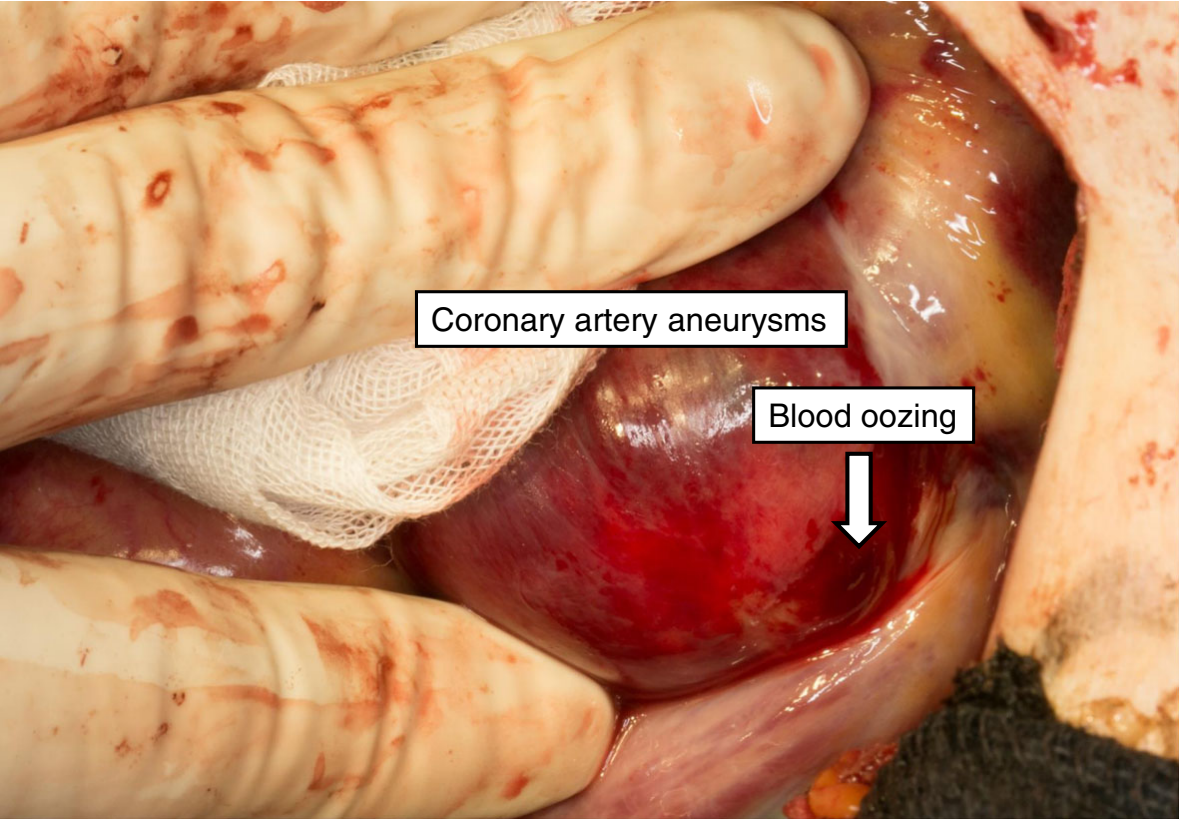
Intraoperative photograph. Blood oozing (arrow) on the wall of the giant coronary artery aneurysm

The patient was transferred to our hospital for surgical treatment. Cardiac tamponade due to either rupture of CAA or CAVF was diagnosed, and emergency surgery was performed through a median sternotomy. The pericardial sac contained blood and clots. On completion of the pericardiotomy, blood oozing was evident and a rupture site was located on the wall of the giant CAA, originating from the ostium of RCA (Figs. 3 and 4). Cardiopulmonary bypass was established via an ascending aortic cannula and bicaval cannula. The patient was cooled to moderate hypothermia, the aorta was cross-clamped, and cold blood cardioplegic solution was delivered. The giant CAA was opened and the contents were evacuated. Suture closure of the inflow and outflow of the aneurysm was carried out, and the aneurysmal cavity was obliterated by multiple sutures. After surgery for the giant CAA, the second biggest CAA was found next to the giant CAA and measured 20 mm in diameter. This CAA was treated using a similar maneuver. No further giant aneurysms were subsequently recognized. Coronary artery bypass grafting was not performed, because the aneurysms were located in a small branch of the coronary artery. The smaller aneurysm of the LAD was not operated on, because this aneurysm was < 10 mm in diameter and this was not thought to represent a site of rupture. Furthermore, the fistula was not ligated because of the predicted difficulty of reconstruction.

**Fig. 5:**
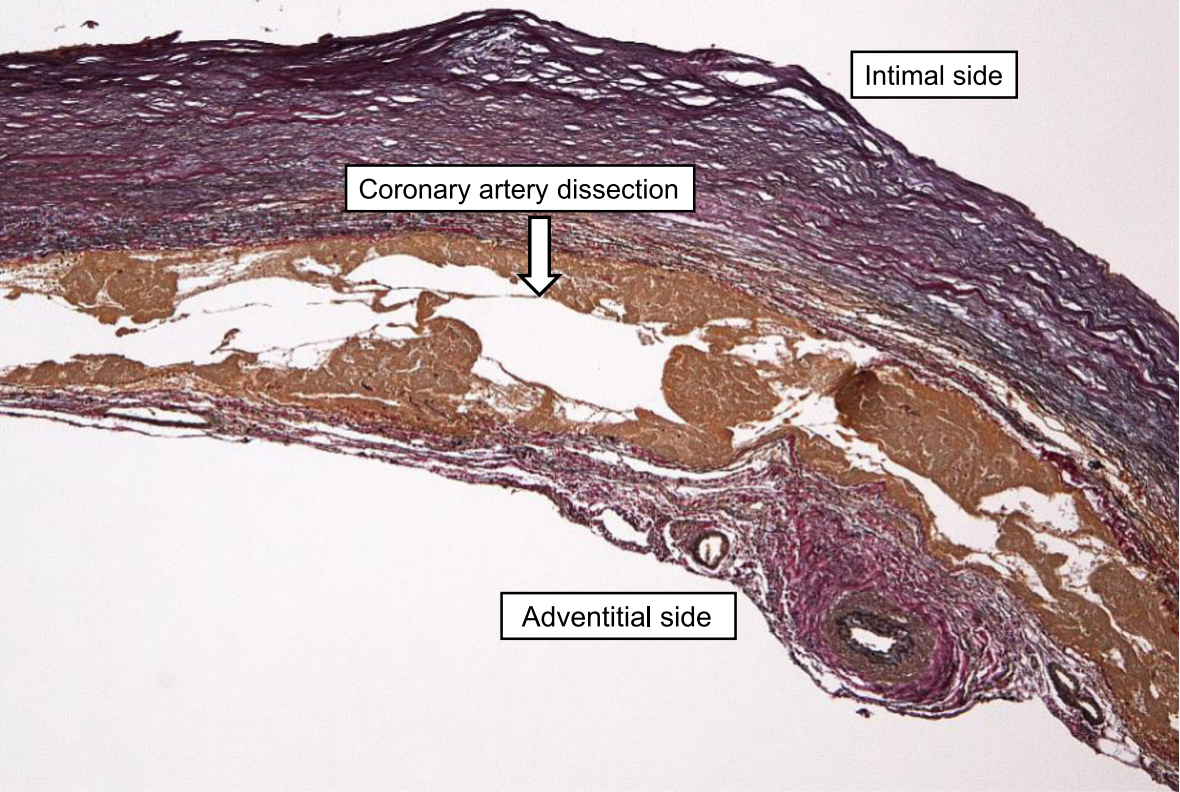
Histopathological findings of the excised coronary artery aneurysm. Coronary artery dissection (arrow) is evident. Elastica van Gieson-stained section

The patient made an uneventful recovery and was discharged from hospital on postoperative day 13. A histopathological assessment of the excised CAA revealed coronary artery dissection (Fig. 5). Follow-up CT was performed every 1 year after surgery. The patient remained well as of the 4-year follow-up, showing no expansion of the small coronary aneurysm.

## Discussion

CAA is quite rare, occurring in 0.15–4.9% of patients undergoing coronary angiography [1, 2]. Giant CAA is even more infrequent. This abnormality is thought to originate in different ways. Atherosclerosis is the chief cause of CAA in adults, followed by Kawasaki disease. Other underlying conditions include connective tissue disease, arteritis, and CAVF [3], defined as an abnormal communication between a coronary artery and a cardiac chamber, great artery, or vena cava. Dilatation of the CAVF is common, and although 19% of these may become aneurysmal [4], rupture of the aneurysm appears very rare. CAA with CAVF > 30 mm in diameter is at risk of rupture [5]. CAA > 50 mm in diameter shows an association with CAVF in 21.4% of cases [6]. That is, one of the causes of giant CAA is CAVF. In the present case, we considered that these CAAs derived from CAVF.

Coronary angiography is essential for establishing the diagnosis of CAA with CAVF. Newer imaging modalities, such as multidetector-row CT (MDCT), can be useful for demonstrating CAVF. MDCT is also well suited to noninvasively evaluating communications between CAA and the vessels in CAVF [7]. In the present case, CT was undertaken at a nearby hospital, not our hospital. MDCT was therefore not performed in this case.

Acute cardiac tamponade is often caused by acute aortic dissection, free wall rupture due to acute myocardial infarction, or a traumatic thoracic accident. The present experience indicates that rupture of CAA with CAVF should be considered as one potential cause of acute cardiac tamponade. Few reports have described ruptured CAA with CAVF resulting in cardiac tamponade [2, 8]. A review of published reports described the risk factors for rupture of CAA with CAVF as female gender, saccular aneurysm, Asian ethnicity, origin of the aneurysmal fistula in the left coronary artery, and hypertension [9].

We have here reported a case of ruptured CAA with CAVF in which the patient developed presyncope, back pain, and subsequent cardiac tamponade, but emergent surgical treatment was successfully performed.

We started to perform emergency surgery for aneurysm excision, and blood was observed oozing from the boundary around the giant CAA of the RCA and CAVF. Ligation of the fistula and excision of the giant aneurysm were initially considered necessary, but were not performed because the aneurysm of the LAD was < 10 mm in diameter. The patient remained well at the 4-year follow-up with no expansion of the small coronary aneurysm. Previous reports of ruptured CAA have noted diameter over 30 mm [2, 10]. Furthermore, CAA and CAVF in this case did not cause other symptoms such as angina or dyspnea. Complete surgical repair, including exclusion of the smaller LAD aneurysm and coronary revascularization to a graftable branch in the left anterior descending area combined with ligation of the associated fistula, is quite challenging and sometimes fatal because of the risk of broad anterior myocardial infarction without revascularization due to the lack of graftable branches. Accordingly, only the two giant CAAs of the RCA were operated on.

The 5-year survival rate of patients with aneurysms was 71% in one report that defined CAA as an aneurysm over double the size of the normal coronary artery or more than 8 mm in diameter [11]. We consider careful follow-up as important for this case.

## Conclusion

We successfully performed surgical treatment for a ruptured giant CAA. On the basis of this case, we propose that rupture of a CAA should be considered as one cause of cardiac tamponade.

## Abbreviations

CAA: Coronary artery aneurysm
CAVF: Coronary arteriovenous fistula
CT: Computed tomography
LAD: Left anterior descending artery
MDCT: Multidetector-row CT
RCA: Right coronary artery

## References

[1] Li D, Wu Q, Sun L, Song Y, Wang W, Pan S (2005). Surgical treatment of giant coronary artery aneurysm. J Thorac Cardiovasc Surg.

[2] Kimura S, Miyamoto K, Ueno Y (2006). Cardiac tamponade due to spontaneous rupture of large coronary artery aneurysm. Asian Cardiovasc Thorac Ann.

[3] Holinski S, Dohmen PM, Lembcke A, Konertz W (2009). Surgical management of multiple coronary artery aneurysms, including the giant form. Tex Heart Inst J.

[4] Urrutia-S CO, Falaschi G, Ott DA, Cooley DA (1983). Surgical management of 56 patients with congenital coronary artery fistulas. Ann Thorac Surg.

[5] Takeucih N, Takada M, Nishibori Y, Maruyama T (2012). A case report of coronary arteriovenous fistulas with an unruptured coronary artery aneurysm successfully treated by surgery. Case Rep Cardiol.

[6] Keyser A, Hilker MK, Husser O, Diez C, Schmid C (2012). Giant coronary aneurysms exceeding 5 cm in size. Interact Cardilvasc Thorac Surg.

[7] Hara H, Moroi M, Araki T, Kunimasa T, Tsunoda T, Suzuki M (2005). Coronary artery fistula with an associated aneurysm detected by 16-slice multidetector row computed tomographic angiography. Heart Vessel.

[8] Harada Y, Mori A, Abiko T, Saka S, Shinagawa T, Yoshimoto T (2013). Cardiac tamponade due to the rupture of the coronary artery fistula. Cardiovasc Diagn Ther.

[9] Said SA, Schroeder-Tanka JM, Mulder BJ (2008). Female gender and the risk of rupture of congenital aneurysmal fistula in adults. Congenit Hearts Dis.

[10] Vijayanagar R, Shafii E, DeSantis M, Waters RS, Desai A (1994). Surgical treatment of coronary aneurysms with and without rupture. J Thorac Cardiovasc Surg.

[11] Baman TS, Cole JH, Devireddy CM, Sperling LS (2004). Risk factors and outcomes in patients with coronary artery aneurysms. Am J Cardiol.

